# Supplementary material for: Causal motifs and existence of endogenous cascades in directed networks with application to company defaults
Source: Sci Rep. 2021 Dec 15;11:24028. doi: 10.1038/s41598-021-02976-8 (PMC8674357; doi:10.1038/s41598-021-02976-8)
Supplement: Supplementary file 1 — Supplementary Information. [file 41598_2021_2976_MOESM1_ESM.pdf]

Supplementary Information for: Causal motifs and existence of endogenous cascades in directed networks with application to company defaults

Irena Barjašić[1], Hrvoje Štefančić[2], Vedrana Pribičević[3,4], and Vinko Zlatić[5]

[1]Faculty of Science, University of Zagreb, 10000 Zagreb, Croatia
[2]Catholic University of Croatia, Ilica 242,10000 Zagreb, Croatia
[3]Zagreb School of Economics and Management, Zagreb, Croatia
[4]Faculty of Economics, University of Ljubljana, Ljubljana, Slovenia
[5]Division of Theoretical Physics, Rudjer Bošković Institute, Zagreb, Croatia

September 28, 2021

1 Data description and network construction

The data was web scraped from the website of the Croatian Financial Agency, which publicly discloses all documents related to the Chapter 11 type bankruptcies, which involved debt renegotiation and restructuring in the Republic of Croatia. The website features all available documents for each company in the pre-bankruptcy settlement. A new law was passed at the end of 2012, which defined the criteria companies had to meet in order to file for this type of bankruptcy: failing to attain liquidity over the course of 60 days and at most 21 days passing since their insolvency onset. The types of companies which could file for this procedure include a public limited company, a limited liability company or a sole proprietorship. When filing for the procedure, each company had to officially report an extensive list of its creditors to the Croatian Financial Agency on the day they filed for the procedure. The agency then published the scanned documents with creditor lists on their website, usually in the form of PDF files. We web scraped two key files; data was gathered first from the restructuring plan document, which contained an initial list of creditors, and this list was then compared to list of creditors stated in the court approved settlement document, which was issued at the end of the procedure. This was done to account for discrepancies between the initial list of creditors and the final list of creditors; some creditors were omitted by the debtor and were added later in the procedure, while other creditors may have received payment during the duration of the settlement. In the end we were left with lists which only recorded de iure debt for every creditor in a single point in time for every firm finishing the procedure. In other words, we only included the debt to creditors that was not settled at the end of the procedure, but was to be renegotiated and reprogrammed through negotiations between the debtor and the creditors.

Despite the data being public, much effort was invested into wrangling and cleaning the data. Firstly, all web scraped PDF files were converted into Excel files and tables containing information on creditors were extracted into a separate, orderly Excel file. Six student research assistants from Zagreb School of Economics and Management did this manually for every firm, pasting the data into an Excel form that was pre-approved by the researchers that closely supervised them. Ambiguous data points were referred back to the researchers, which jointly decided how to systematically resolve them. Most such cases had either missing data on creditors or an inconsistent amount of debt reported across two scanned documents used for reference. Upon completing their work and the final control of the forms containing the data and the PDF documents by the researchers to assure accuracy, students were financially compensated. For every subject in the procedure, name, address and tax identification number were gathered for both the debtor and all the creditors, together with the starting date of the procedure. The amount of debt for each creditor was gathered as well. Since having accurate data was key to network construction, we employed several additional steps to minimize potential errors. The main source of errors were false tax identification numbers or names of creditors. Firstly, we compared tuples containing tax identification numbers and names of creditors to see whether creditors with the same name have different identification numbers or if two different creditors shared the same identification number. Secondly, we compared all the amounts of debts to search for potential duplicates. Thirdly, we singled out creditors with zero debt or debtors who had themselves listed as creditors. Lastly, we found all unique foreign creditors that did not have an identification number and assigned them a special, shorter identification number. All of the data anomalies were inspected manually and the errors were corrected before the network was constructed.

The data we finally obtained spans from December 19th 2012 to February 26th 2014, and contains 25469 creditors and debtors, and 52507 debts, where debtors are exclusively firms and creditors range from banks, private and public firms, government and individuals. Total amount of reported debt was 5.97 billion euros, which corresponded to 13,6 percent of Croatia's GDP in 2014. The choice of specific time frame for dataset was intentional; in order to observe cascades, data collection began when the largest and most interconnected debtors filed for the procedure soon after the law was introduced at the end of 2012, following the initial illiquidity build-up, while number and size of firms significantly decreased in the next two years. Additionally, we only include recession years to avoid business cycle effects of economic recovery which may exogenously affect probabilities of defaults. i.e. may exert a different field effect on our network.

From the final form of the data, which is a table with every row representing a debt from a debtor to a creditor and containing all the aforementioned information, we use the information on the debtor (name, address and personal identification number), the creditor (name, address and personal identification number) and the starting time of the pre-bankruptcy settlement. As we are studying the propagation of default, we employ only the firms that are both debtors and creditors, i.e. that can both have their default caused by their debtor's default, and propagate their own default into the network. We filter these firms by making an intersection of their tax identification numbers, and represent them as vertices. To each vertex we attribute the starting time of the pre-bankruptcy settlement as the time of default, and for every debt (from the debtor to their creditor) create a directed edge in the stated direction. The scheme of the network creation is depicted in Fig. S1.

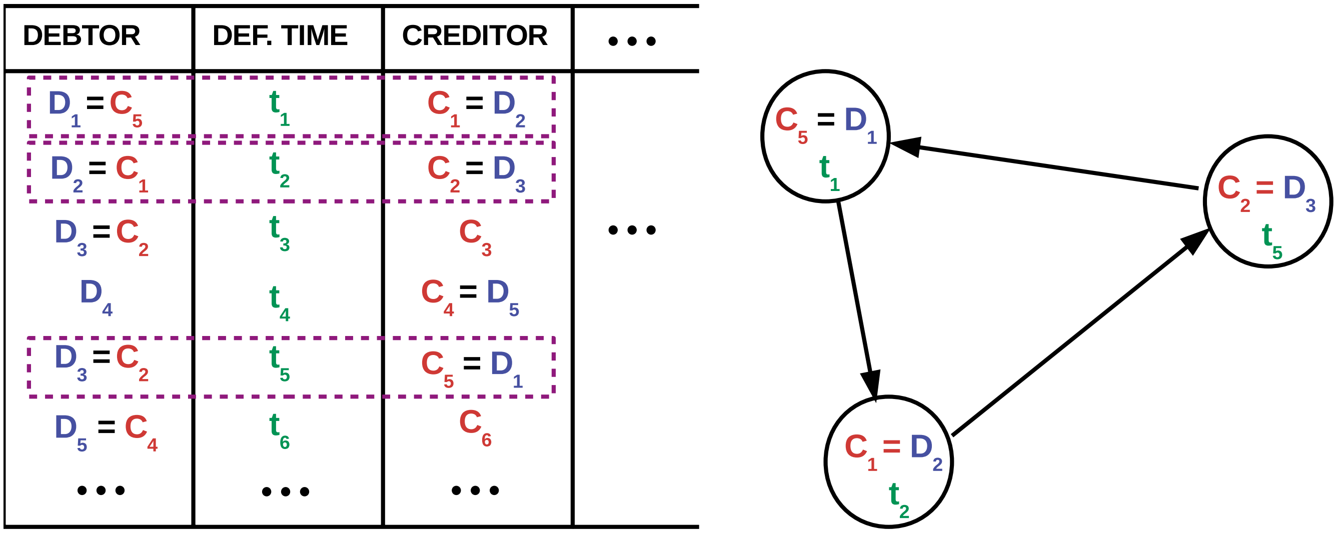

Figure 1. The scheme of the network construction from the data. On the left side of the figure we show a simplified table with the three columns that we use from the data; the identities of debtors, the identities of creditors and the start of the pre-bankruptcy settlement (default time). We mark with the purple dashed rectangles the rows that satisfy the requirement that the firms we use have to be both debtors and creditors. Those rows are used in the construction of the network on the right, the debt from the debtor to the creditor is represented with a directed edge from the vertex that entered the settlement as a debtor to the vertex that entered the settlement as a creditor, and the time of default is attributed to the vertex that entered the settlement as a debtor.

2 Expected frequency of the motifs

Since our method revolves around using simulations to perturb timestamps in data, it is instructive to first consider what frequency of the motifs would one expect if only the exogenous process changed the state of vertices in the network. In that scenario, the probability of finding each individual casual motif $\mathscr{C}\mathscr{M}$ would be proportional to *(i)* probability $p(\mathscr{M})$ of finding the structural motif $\mathscr{M}$ on which a causal motif could develop; *(ii)* probability $\mathscr{P}(T)$ that all the vertices of the given motif $\mathscr{M}$ have changed their state (got infected, have defaulted) by the time T; and *(iii)* the probability that the vertices in the motif $\mathscr{M}$ have defaulted in such an order that all the edges of the motif have become causal.

In this exercise, the third probability is of interest to us. It can be computed using simple combinatorics, because only the mutual ordering of timestamps is of importance, and not any other details of the probability distribution of those timestamps. For example, for the third motif in the leftmost column of table, all that is important is that t_1 is the earliest timestamp out of the three timestamps and that occurs in $1/3$ of the random realizations.

Causal Motif	Probability	Causal Motif	Probability
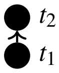 $t_1 < t_2$	$p(\mathscr{M})\frac{1}{2}\pi(T)^2$	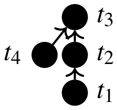 $t_1 < t_2 < t_3$; $t_4 < t_3$	$p(\mathscr{M})\frac{1}{8}\pi(T)^4$
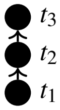 $t_1 < t_2 < t_3$	$p(\mathscr{M})\frac{1}{6}\pi(T)^3$	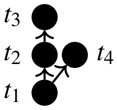 $t_1 < t_2 < t_3$; $t_1 < t_4$	$p(\mathscr{M})\frac{1}{8}\pi(T)^4$
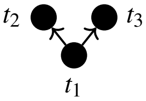 $t_1 < t_2, t_3$	$p(\mathscr{M})\frac{1}{3}\pi(T)^3$	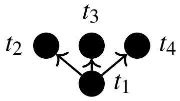 $t_1 < t_2, t_3, t_4$	$p(\mathscr{M})\frac{1}{4}\pi(T)^4$
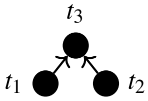 $t_1, t_2 < t_3$	$p(\mathscr{M})\frac{1}{3}\pi(T)^3$	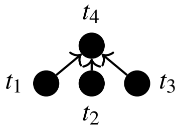 $t_1, t_2, t_3 < t_4$	$p(\mathscr{M})\frac{1}{4}\pi(T)^4$
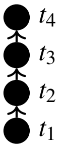 $t_1 < t_2 < t_3 < t_4$	$p(\mathscr{M})\frac{1}{24}\pi(T)^4$	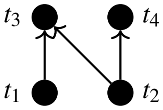 $t_1, t_2 < t_3$; $t_2 < t_4$	$p(\mathscr{M})\frac{5}{24}\pi(T)^4$
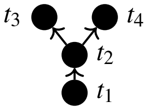 $t_1 < t_2 < t_3, t_4$	$p(\mathscr{M})\frac{1}{12}\pi(T)^4$	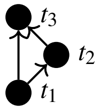 $t_1 < t_2 < t_3$	$p(\mathscr{M})\frac{1}{6}\pi(T)^3$
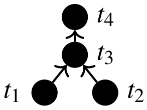 $t_1, t_2 < t_3 < t_4$	$p(\mathscr{M})\frac{1}{12}\pi(T)^4$		

Table 1. Table with all possible causal motifs up to order 3. Their probability represents the probability of occurrence of such a motif if only the exogenous influence exists.

In order to compute that probability for higher order motifs than the one presented here, we found that integrating the exogenous probability over time is a simpler approach. Let us suppose that the exogenous probability per unit time of the change of state of an individual vertex is $\phi(t)$. Then we can write an equation for the probability of a random appearance of the three-edge causal motif that looks like letter N in Supplementary Table S1 as:

$$\begin{aligned}
P(\mathscr{C}\mathscr{M}) &= p(\mathscr{M})\int_0^T\int_0^{t_3}\left(\int_0^{t_3}\phi(t_1)dt_1\int_{t_2}^{T}\phi(t_4)dt_4\right)\phi(t_2)\phi(t_3)dt_2dt_3 \\
&= p(\mathscr{M})\int_0^T\int_0^{t_3}\pi(t_3)[\pi(T)-\pi(t_2)]\phi(t_2)dt_2\phi(t_3)dt_3 \\
&= p(\mathscr{M})\frac{5}{24}\pi(T)^4, \qquad (1)
\end{aligned}$$

where we defined $\pi(\tau) := \int_0^\tau \phi(t)dt$, so the relation between cumulative probability and probability density is $\int \phi(t)dt \equiv \int d\pi(t)$.

As we see in Supplementary Table S1, the higher the order of a motif is, the less probable it is to be found in a purely exogenous process. We will use the count for each motif order $\mathscr{N}(t)$ at time t as the statistic to test if the data can be explained only through exogenous process, by comparing it against the RRM.

3 The number of vertices defaulted by the exogenous and the endogenous process components

In order to show the difference between ζ that we used as a control parameter of the processes and the actual number of vertices that defaulted through the endogenous or the exogenous process, we write rate equations using the mean field approximation. Using those equations we compute how many vertices n_α default through the exogenous component of the process and how many vertices n_β default through the endogenous process in an Erdős-Rényi network with N vertices, and average in- and out-degrees $\langle k_i \rangle$ and $\langle k_o \rangle$.

The increase of the number of exogenously defaulted vertices, dn_α, is proportional to the number of the non-defaulted vertices $N - n_\alpha - n_\beta$, rate α and time increment dt

$$dn_\alpha = (N - n_\alpha - n_\beta)\alpha dt. \tag{2}$$

The increase of the number of endogenously defaulted vertices dn_β is proportional to the number of defaulted vertices $n = n_\alpha + n_\beta$, and their average out degree $\langle k_o \rangle$, which gives the number of edges through which the default can propagate. For the propagation to occur, the vertices at the ends of those edges have to be previously non-defaulted, which can be modeled with the mean field probability that the vertex is not defaulted $1 - \frac{n_\alpha + n_\beta}{N}$. Together with the rate β and time increment dt, all these factors lead to the equation:

$$dn_\beta = (n_\beta + n_\alpha)\langle k_o \rangle (1 - \frac{n_\alpha + n_\beta}{N})\beta dt. \tag{3}$$

From the equations (2) and (3), one can easily extract the time dependence in order to obtain the "phase space" differential equation, using the fact that both n_α and n_β are monotonously increasing functions of time.

$$\frac{dn_\alpha}{dn_\beta} = \frac{N\zeta}{\langle k \rangle_o (n_\alpha + n_\beta)} \tag{4}$$

$$dn_\beta = \frac{\langle k \rangle_o}{N\zeta}(n_\alpha dn_\alpha + n_\beta dn_\alpha) \tag{5}$$

The equation (5) is a linear equation that can easily be integrated to obtain:

$$n = \frac{N\zeta}{\langle k \rangle_o}\left(e^{\frac{\langle k \rangle_o n_\alpha}{N\zeta}} - 1\right) \tag{6}$$

$$\frac{n_\alpha}{n_\beta} = \frac{\frac{\zeta}{\langle k \rangle_o} ln(\frac{d\langle k \rangle_o}{\zeta} + 1)}{d - \frac{\zeta}{\langle k \rangle_o} ln(\frac{d\langle k \rangle_o}{\zeta} + 1)}, \tag{7}$$

where $n = n_\alpha + n_\beta$ is a total number of defaulted vertices in the network and $d = n/N$ is the total default percentage in the network with N vertices. For the case of VM, we substitute $\zeta \to \zeta \langle k_i \rangle$, and, since $\langle k_i \rangle = \langle k_o \rangle$, the equations are

$$n = N\zeta\left(e^{\frac{n_\alpha}{N\zeta}} - 1\right) \tag{8}$$

$$\frac{n_\alpha}{n_\beta} = \frac{\zeta ln(\frac{d}{\zeta} + 1)}{d - \zeta ln(\frac{d}{\zeta} + 1)}, \tag{9}$$

From equations (7) and (9) it is clear that for a given ζ and for $\langle k_o \rangle > 1$, the VM process will always have a higher ratio n_α / n_β than the SI process. It is also clear that this ratio is monotonously decreasing with respect to the total default percentage d. Note that for voter model, the ratio n_α / n_β does not depend on the degree of the network, but only on the process parameters and default percentage.

4 Kolmogorov-Smirnov test results

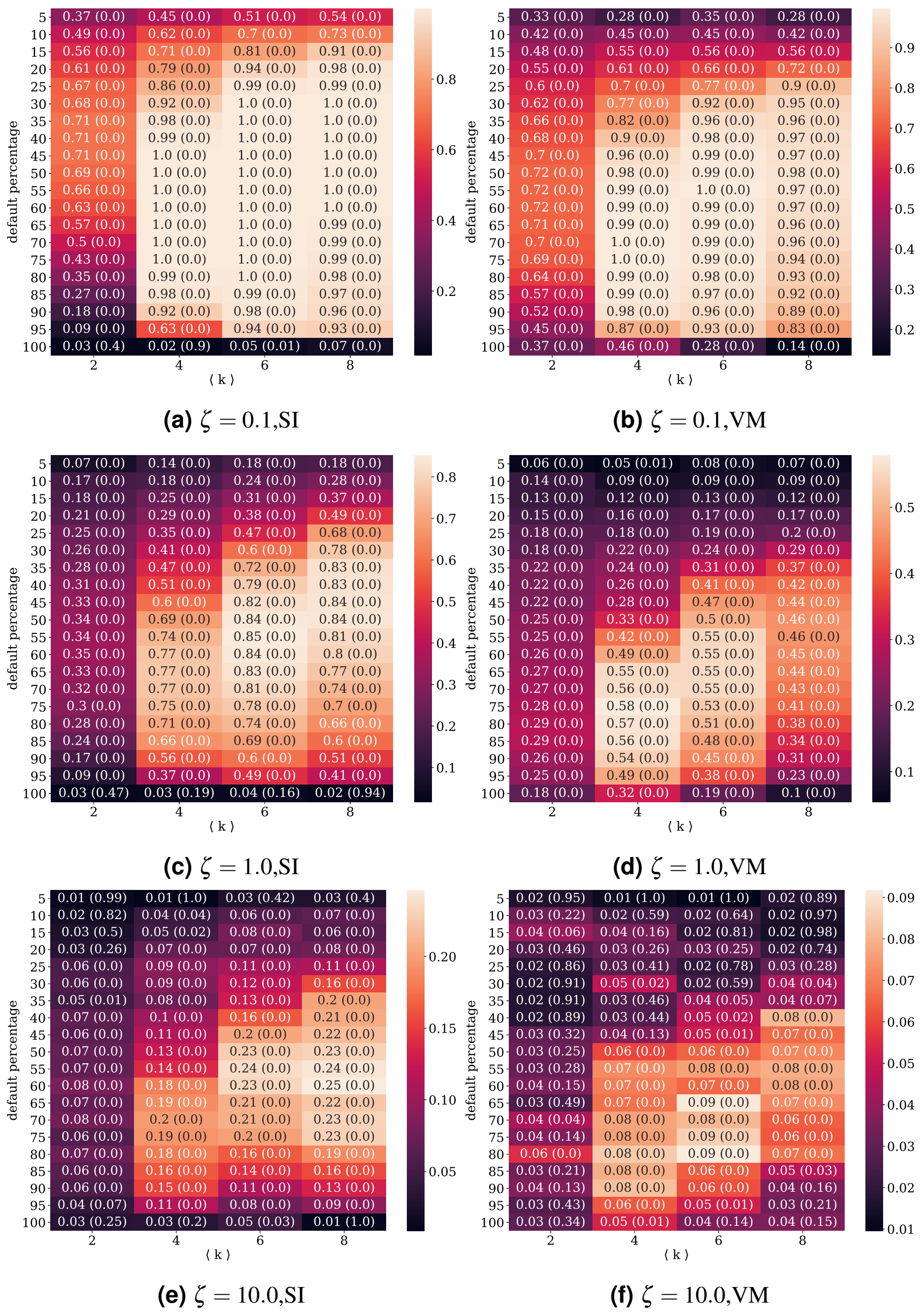

Figure 2. Kolmogorov-Smirnov test results for the largest component statistic. Next to the value of the statistic its p-value is written in brackets. Subfigures **a**), **c)** and **e)** show the results for the SI process, and subfigures **b**), **d)** and **f)** show the results for the voter model process.

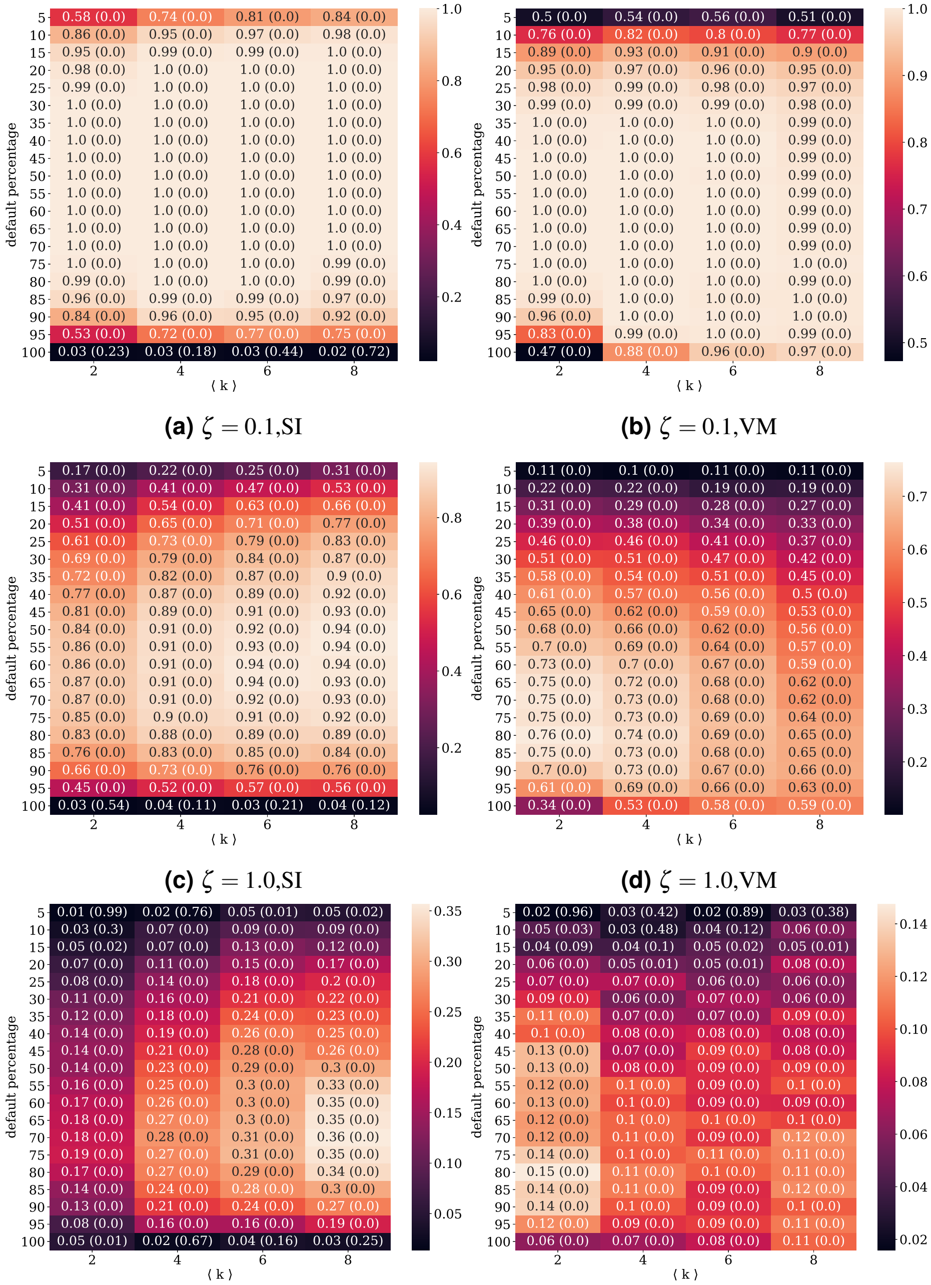

(a) $\zeta = 0.1$,SI **(b)** $\zeta = 0.1$,VM

(c) $\zeta = 1.0$,SI **(d)** $\zeta = 1.0$,VM

(e) $\zeta = 10.0$,SI **(f)** $\zeta = 10.0$,VM

Figure 3. Kolmogorov-Smirnov test results for the one-edge statistic. Next to the value of the statistic its p-value is written in brackets. Subfigures **a**), **c)** and **e)** show the results for the SI process, and subfigures **b**), **d)** and **f)** show the results for the voter model process.

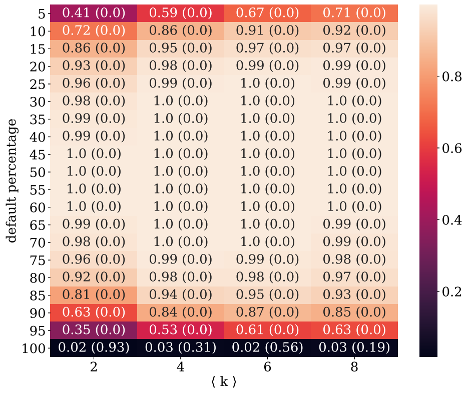

(a) $\zeta = 0.1$,SI

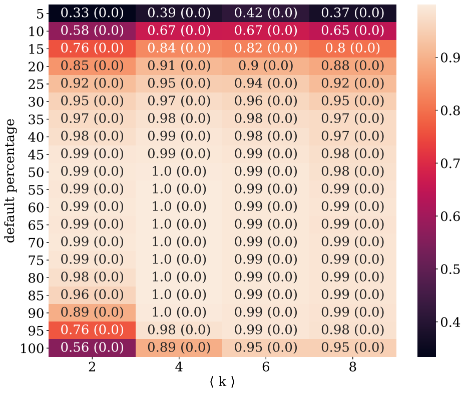

(b) $\zeta = 0.1$,VM

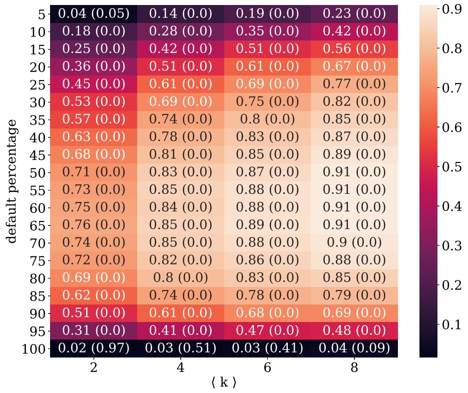

(c) $\zeta = 1.0$,SI

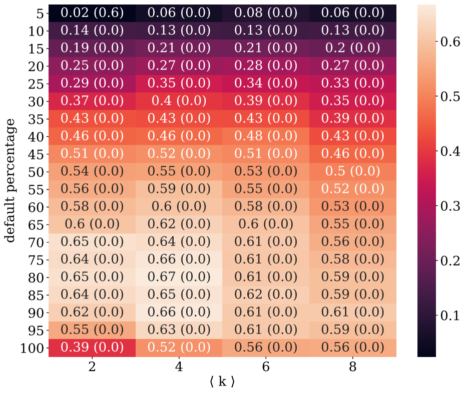

(d) $\zeta = 1.0$,VM

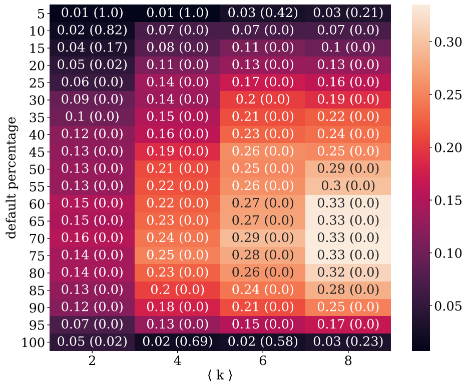

(e) $\zeta = 10.0$,SI

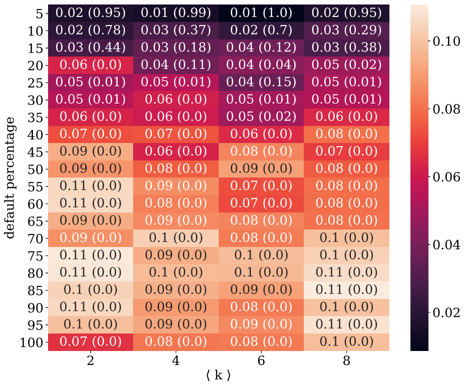

(f) $\zeta = 10.0$,VM

Figure 4. Kolmogorov-Smirnov test results for the two-edge statistic. Next to the value of the statistic its p-value is written in brackets. Subfigures **a)**, **c)** and **e)** show the results for the SI process, and subfigures **b)**, **d)** and **f)** show the results for the voter model process.

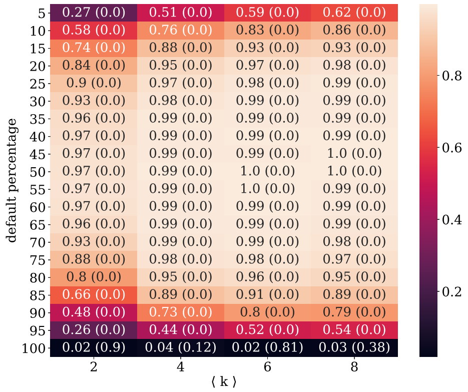

(a) $\zeta = 0.1$,SI

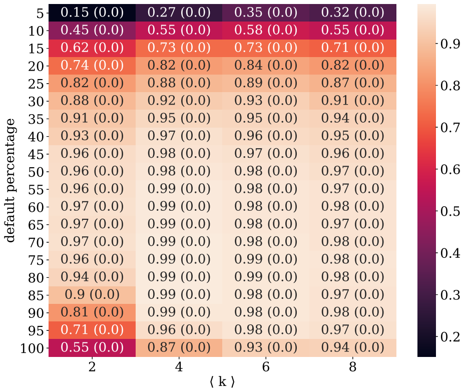

(b) $\zeta = 0.1$,VM

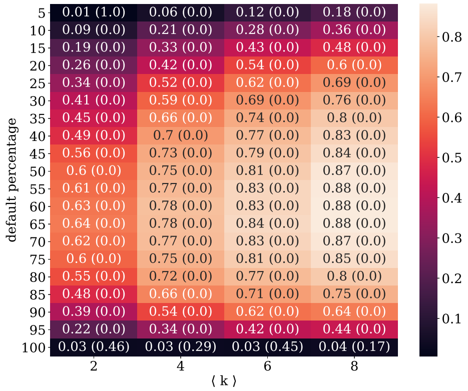

(c) $\zeta = 1.0$,SI

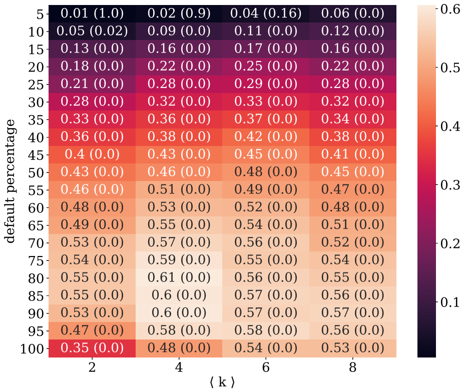

(d) $\zeta = 1.0$,VM

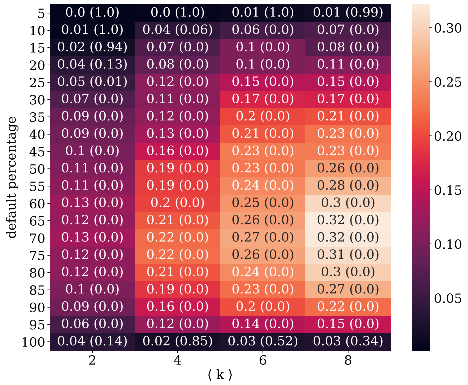

(e) $\zeta = 10.0$,SI

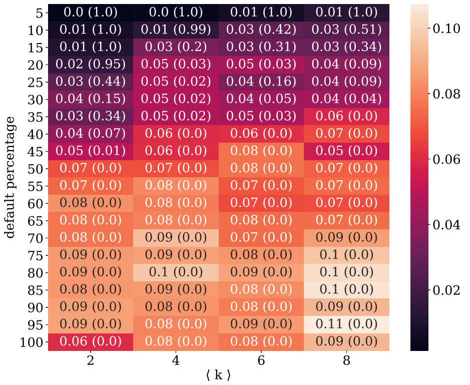

(f) $\zeta = 10.0$,VM

Figure 5. Kolmogorov-Smirnov test results for the three-edge statistic. Next to the value of the statistic its p-value is written in brackets. Subfigures **a)**, **c)** and **e)** show the results for the SI process, and subfigures **b)**, **d)** and **f)** show the results for the voter model process.

5 Z-score

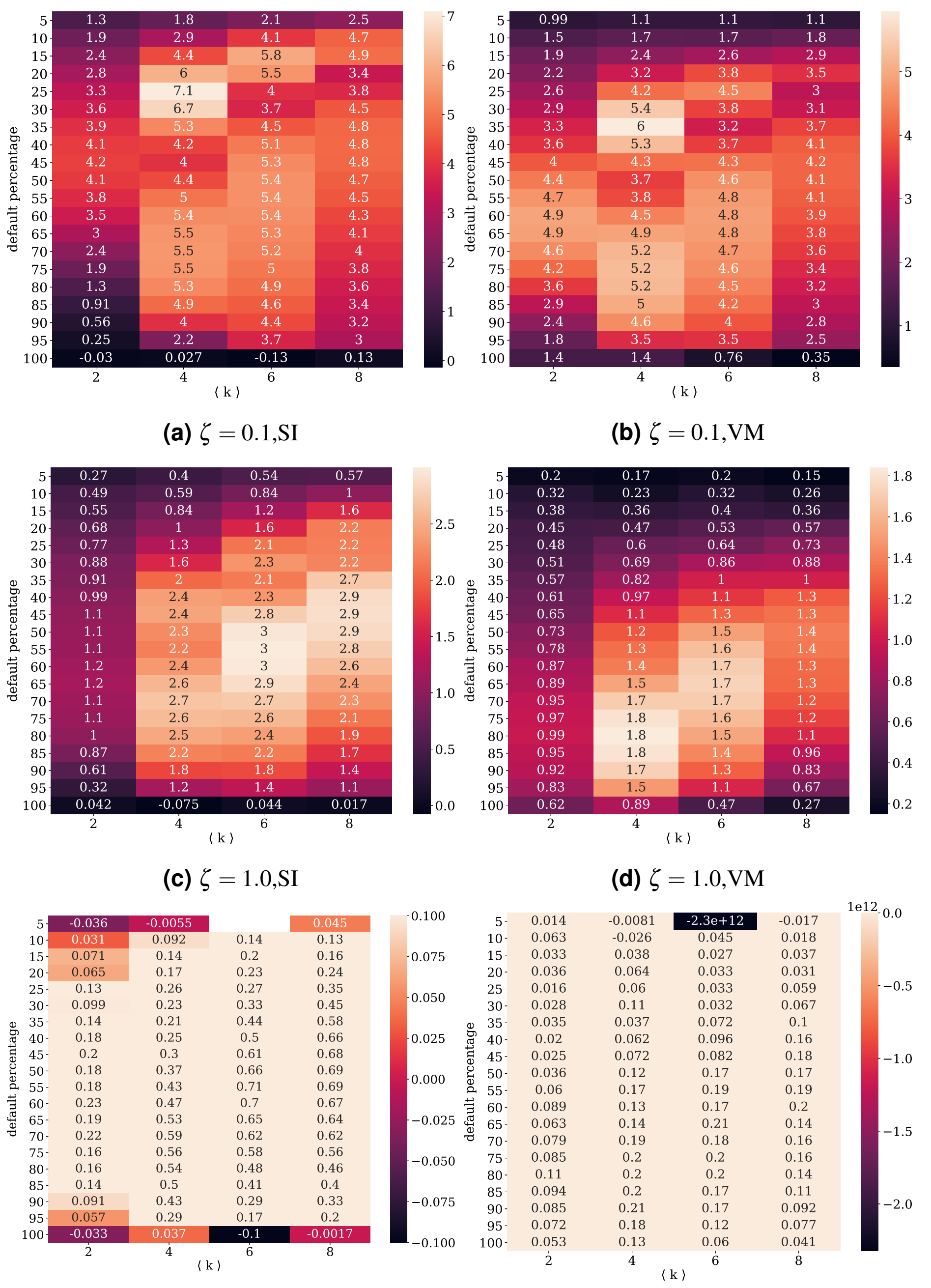

(a) $\zeta = 0.1$,SI **(b)** $\zeta = 0.1$,VM

(c) $\zeta = 1.0$,SI **(d)** $\zeta = 1.0$,VM

(e) $\zeta = 10.0$,SI **(f)** $\zeta = 10.0$,VM

Figure 6. Mean values of Z-scores for the largest component statistic. Subfigures **a)**, **c)** and **e)** show the results for the SI process, and subfigures **b)**, **d)** and **f)** show the results for the voter model process.

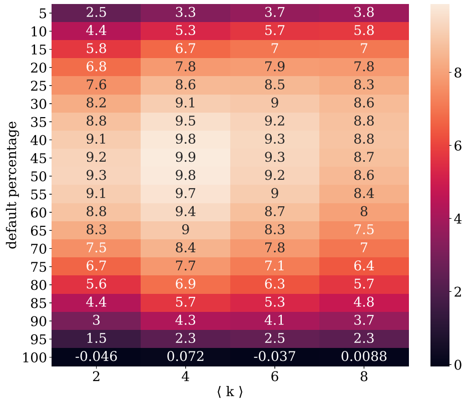

(a) $\zeta = 0.1$,SI

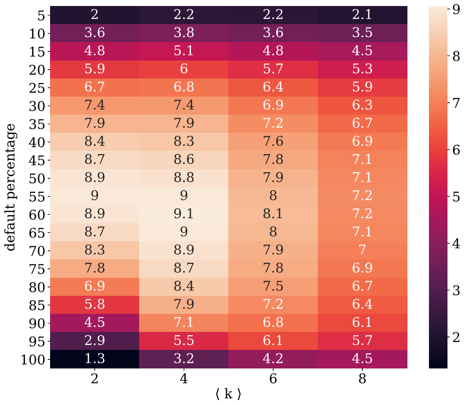

(b) $\zeta = 0.1$,VM

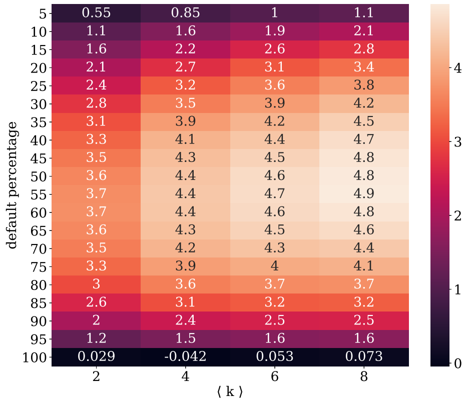

(c) $\zeta = 1.0$,SI

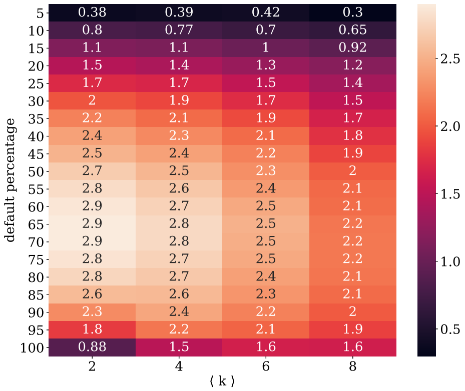

(d) $\zeta = 1.0$,VM

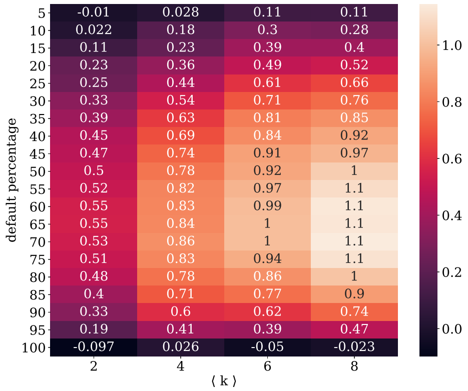

(e) $\zeta = 10.0$,SI

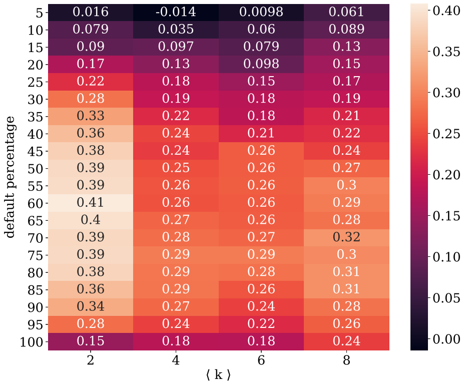

(f) $\zeta = 10.0$,VM

Figure 7. Mean values of Z-scores for the one-edge statistic. Subfigures **a)**, **c)** and **e)** show the results for the SI process, and subfigures **b)**, **d)** and **f)** show the results for the voter model process.

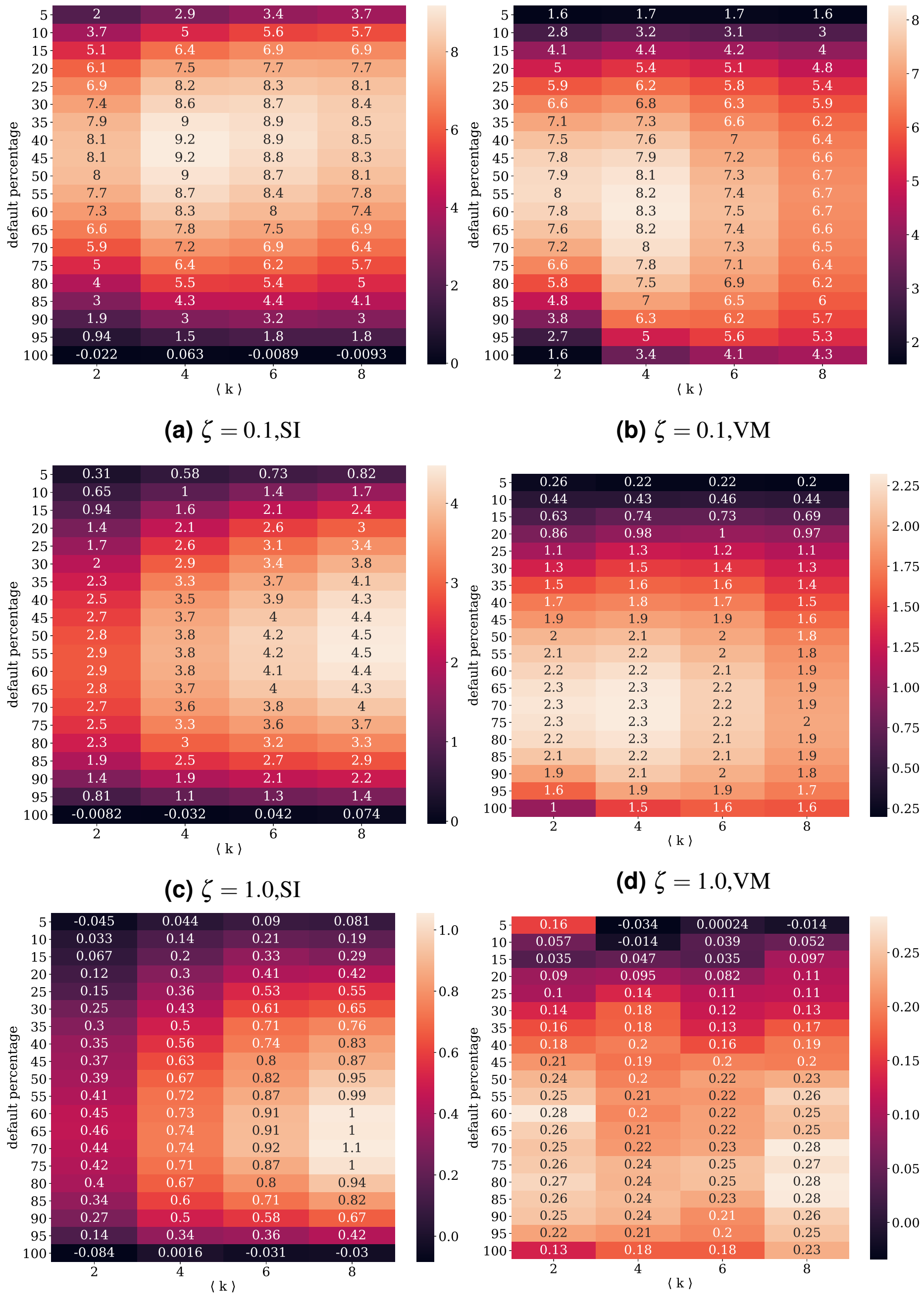

(a) $\zeta = 0.1$,SI

(b) $\zeta = 0.1$,VM

(c) $\zeta = 1.0$,SI

(d) $\zeta = 1.0$,VM

(e) $\zeta = 10.0$,SI

(f) $\zeta = 10.0$,VM

Figure 8. Mean values of Z-scores for the two-edge statistic. Subfigures **a)**, **c)** and **e)** show the results for the SI process, and subfigures **b)**, **d)** and **f)** show the results for the voter model process.

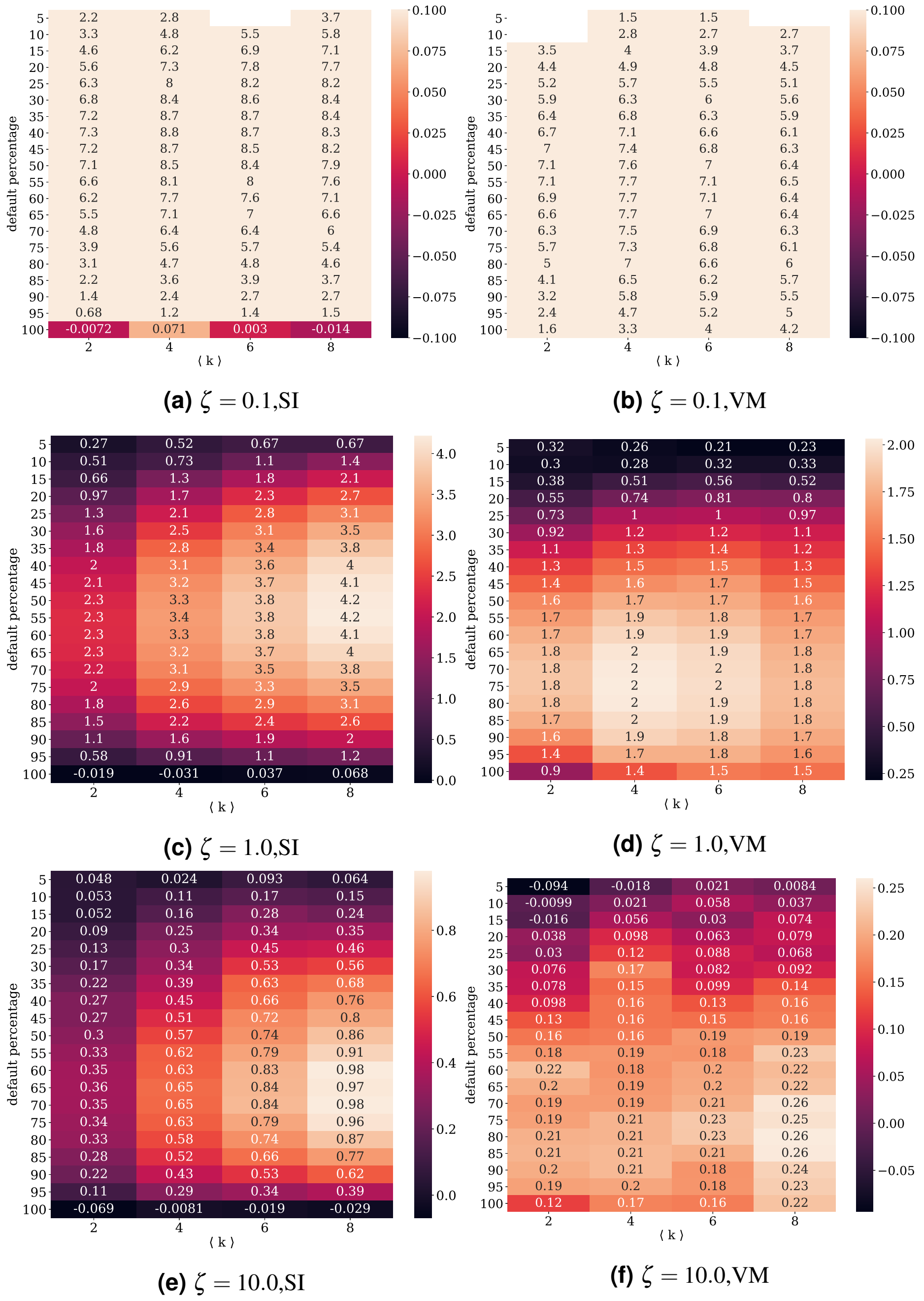

Figure 9. Mean values of Z-scores for the three-edge statistic. Subfigures **a**), **c**) and **e**) show the results for the SI process, and subfigures **b**), **d**) and **f**) show the results for the voter model process.

6 Mahalanobis distance

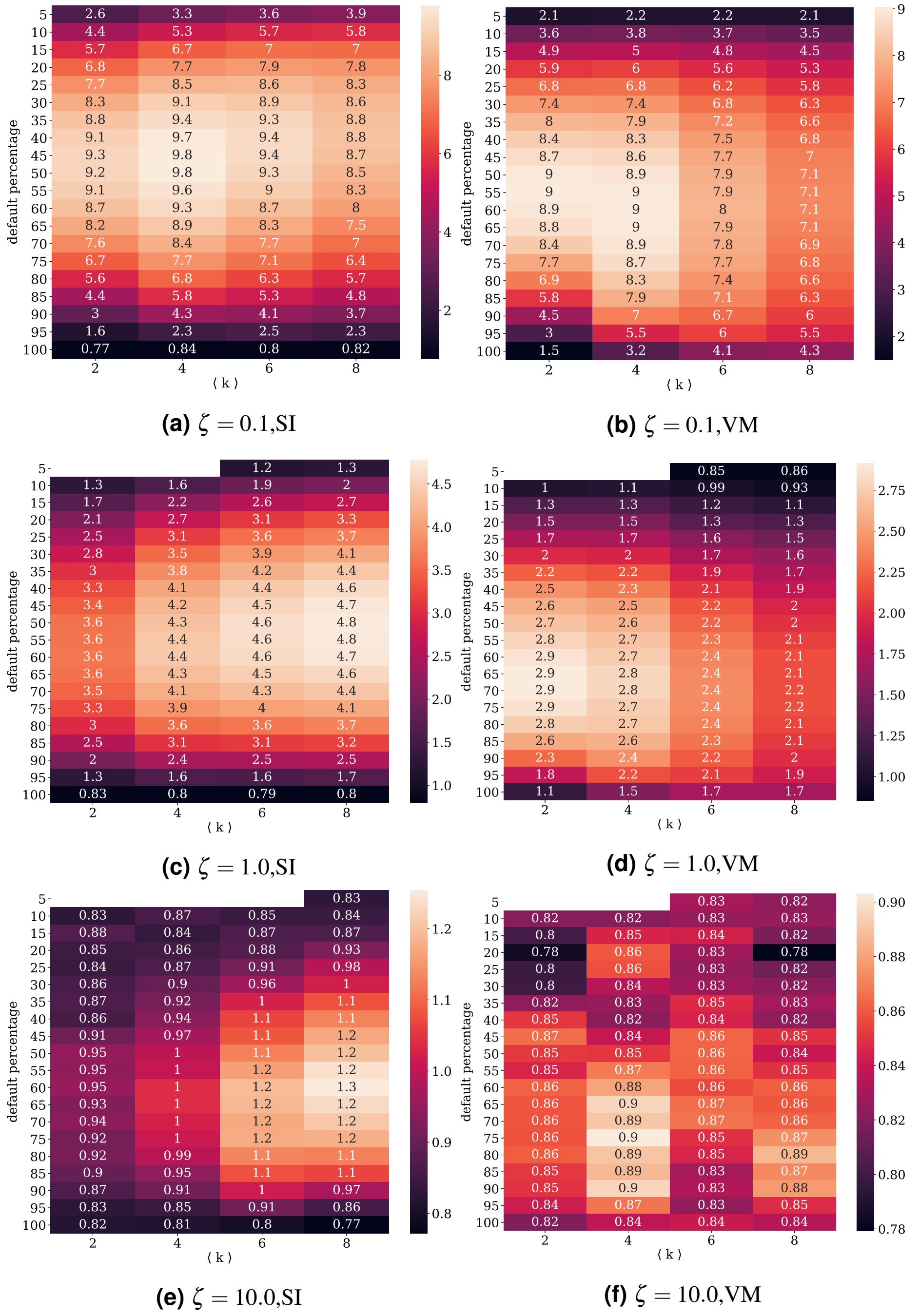

Figure 10. Mean values of Mahalanobis distances for the one-edge statistic. Subfigures **a)**, **c)** and **e)** show the results for the SI process, and subfigures **b)**, **d)** and **f)** show the results for the voter model process.

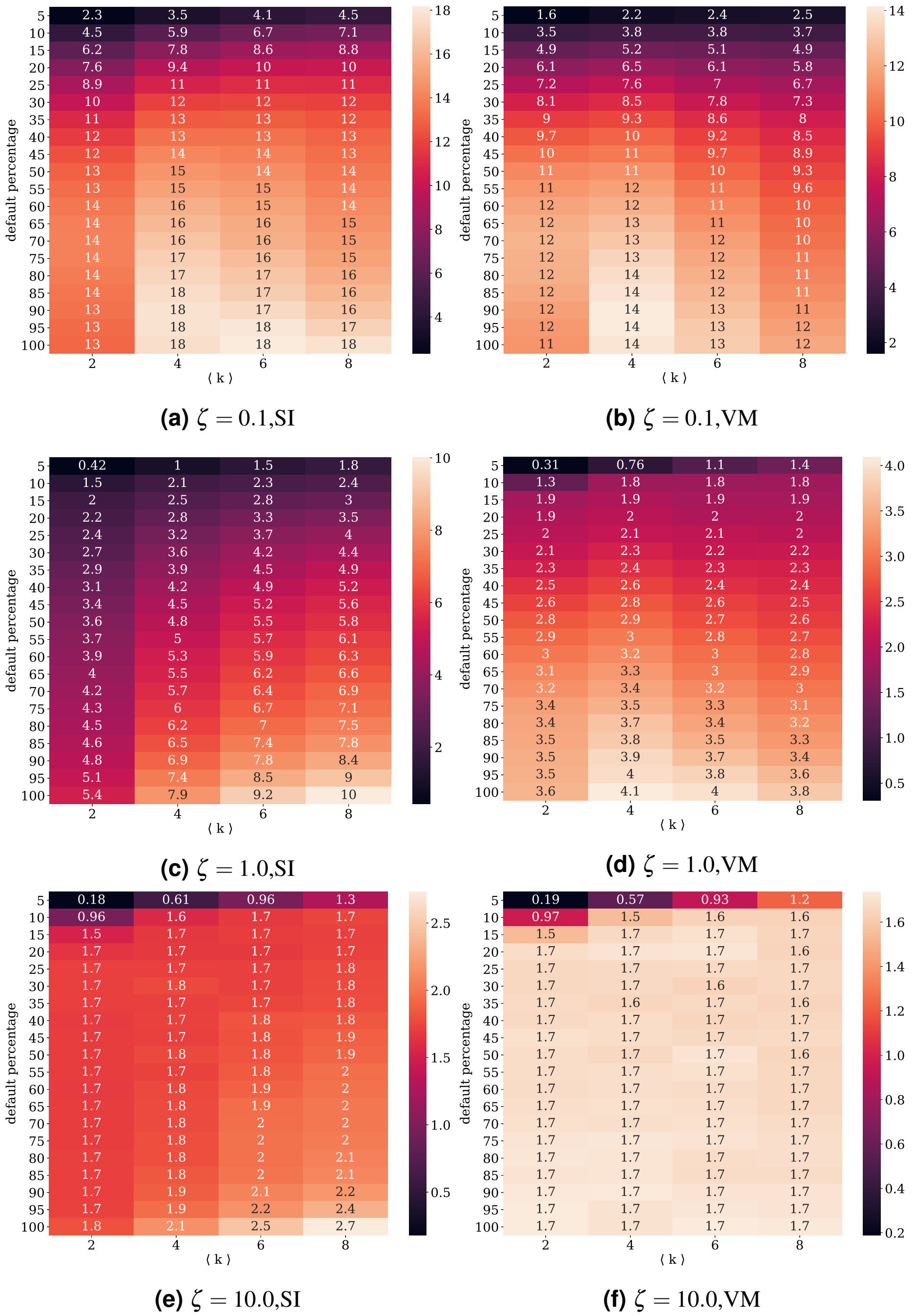

Figure 11. Mean values of Mahalanobis distances for the two-edge statistic. Subfigures **a)**, **c)** and **e)** show the results for the SI process, and subfigures **b)**, **d)** and **f)** show the results for the voter model process.
